# Checked Perspiration

**Published:** 1882-07

**Authors:** 


### CHECKED PERSPIRATION

Is the fruitful cause of sickness, disease and death to multitudes every year. If a tea-kettle of water is boiling on the fire, the steam is seen issuing from the spout, carrying the extra heat away with it, but if the lid be fastened down and the spout be plugged, a destructive explosion follows in a very short time.

Heat is constantly generated within the human body, by the chemical disorganization, the combustion, of the food we eat. There are seven millions of tubes or pores on the surface of the body, which in health are constantly open, conveying from the system by what is called insensible perspiration this internal heat, which having answered its purpose, is passed off like the jets of steam which are thrown from the escape-pipe, in puffs, of any ordinary steam engine; but this insensible perspiration carries with it, in a dissolved form, very much of the waste matter of the system, to the extent of a pound or two or more every twenty-four hours. It must be apparent, then, that if the pores of the skin are closed, if the multitude of valves, which are placed over the whole surface of the human body are shut down, two things take place. First, the internal heat is prevented from passing off, it accumulates every moment, the person expresses himself as burning up, and large draughts of water are swallowed to quench the internal fire—this we call "*Fever.*" When the warm steam is constantly escaping from the body in health, it keeps the skin moist, and there is a soft, pleasant feel and warmth about it. But when the pores are closed, the skin feels harsh, and hot, and dry.

But another result follows the closing of the pores of the skin, and more immediately dangerous ; a main outlet for the waste of the body is closed, it remingles with the blood, which, in a few hours becomes impure, and begins to generate disease in every fiber of the system—the whole machinery of the man becomes at once disordered, and he expresses himself as "*feeling miserable.*" The terrible effects of checked perspiration of a dog, who sweats only by his tongue, is evinced by his becoming "mad." The water runs in streams from a dog's mouth in summer, if exercising freely. If it ceases to run, that is *Hydrophobia*. It has been asserted by a French Physician, that if a person suffering under *Hydrophobia* can be only made to perspire freely, he is cured at once. It is familiar to the commonest observer, that in all ordinary forms of disease, the patient begins to get better, the moment he begins to perspire, simply because the internal heat is passing off, and there is an outlet for the waste of the system. Thus it is that one of the most important means for curing all sickness, is bodily cleanliness, which is simply removing from the mouths of these little pores, that gum, and dust, and oil, which clog them up. Thus it is, also, that personal cleanliness is one of the main elements of health: thus it is, that filth and disease habitate together, the world over.

There are two kinds of perspiration, *sensible* and *insensible*. When we see drops of water on the surface of the body as the result of exercise, or subsidence of fever, that is *sensible perspiration*, perspiration recognized by the sense of sight. But when perspiration is so gentle that it cannot be detected in the shape of water-drops, when no moisture can be felt, when it is known to us only by a certain *softness* of the skin, that is *insensible* perspiration, and is so gentle, that it may be checked to a very considerable extent without special injury. But to use popular language, which cannot be mistaken, when a man is sweating freely, and it is suddenly checked, and the sweat is not brought out again in a very few moments, sudden and painful sickness is a very certain result.

What then checks perspiration? A draft of air while we are at rest, after exercise, or getting the clothing wet and remaining at rest while it is so. Getting out of a warm bed and going to an open door or window, has been the death of multitudes.

A lady heard the cry of fire at midnight: it was bitter cold; it was so near, the flames illuminated her chamber. She left the bed, hoisted the window, the cold wind chilled her in a moment. From that hour until her death, a quarter of a century later, she never saw a well day.

A young lady went to a window in her night-clothes to look at something in the street, leaning her unprotected arms on the stone window-sill, which was damp and cold. She became an invalid, and will remain so for life.

Sir Thomas Colby, being in a profuse sweat one night, happened to remember that he had left the key of his wine cellar on the parlor table, and fearing his servants might improve the inadvertence and drink some of his wine, he left his bed, walked down stairs, the sweating process was checked, from which he died in a few days, leaving six millions dollars in the English funds. His illness was so brief and violent that he had no opportunity to make his will, and his immense property was divided among five or six day laborers who were his nearest relations.

The great practical lesson which we wish to impress upon the mind of the reader is this: *When you are perspiring freely,* KEEP IN MOTION *until you get to a good fire, or to some place where you are perfectly sheltered from any draft of air whatever.*

